# 66. Improving Antimicrobial Stewardship through Allergy Testing Referrals

**DOI:** 10.1093/ofid/ofab466.268

**Published:** 2021-12-04

**Authors:** Clarissa Smith, Victoria Poplin, Brogan Barry, Mathew J Mason, Eric Gregory, Lisa A Clough, Selina Gierer, Adelyn R Newman

**Affiliations:** 1 The University of Kansas Health System, Kansas City, Kansas; 2 University of Kansas Medical Center, Kansas City, Kansas; 3 The University of Kansas School of Medicine, Kansas Cith, Kansas; 4 University of Kansas Health System, Roeland Park, Kansas; 5 The University of Kansas Medical Center, Kansas City, Kansas

## Abstract

**Background:**

Penicillins and cephalosporins (PCN/CEPH) are considered first-line antibiotics for numerous infections for their efficacy, tolerability, and cost effectiveness. Unfortunately, their use may be precluded in approximately 10% of the general adult population who self-report ‘allergy’. As a result, suboptimal antimicrobials are substituted which may increase toxicities, length of hospitalizations, and antimicrobial resistance with subsequent expense and morbidity. Multiple organizations endorse beta-lactam allergy skin testing (BLAST) as an essential component of antimicrobial stewardship programs.^.^In an attempt to better describe this patient population as well as to protocolize and improve rates of referral to allergy/immunology clinic, a quality initiative was undertaken at our institution.

**Methods:**

Adult inpatients for whom an infectious disease consult was placed over a 6-month period were chart-reviewed for PCN/CEPH allergy. Inappropriately charted allergies were reconciled and patients were recommended referral to allergy/immunology for formal evaluation with BLAST when appropriate. Referrals were placed for agreeable patients who were then evaluated for appropriateness through history and then scheduled for BLAST. Patients who tolerated oral exposures without adverse effects had the allergy removed from their chart and were educated.

**Results:**

322 patients met inclusion criteria for allergy referral. Of those, 103 agreed to further evaluation, and referrals were placed for 100%. Unfortunately, 7 patients died before referrals could be completed, and 88 referred patients did not complete BLAST for other reasons. In total 8 patients completed BLAST, and allergy was de-labeled in 75% (N= 6) of those cases.

**Conclusion:**

Our data indicated similar prevalence of reported PCN/CEPH allergy between our institution and the general population. We achieved our aim of improving allergy referral rates among this population, however there was a high rate of attrition in the transitions of care. Qualitative review of selected patients highlights common thematic barriers including the COVID-19 pandemic, fiscal concerns, and acuity of condition. Future directions should include BLAST at the point of care or making referrals from the primary care setting.

**Disclosures:**

**Lisa A. Clough, MD**, **Merck** (Scientific Research Study Investigator)**Merck** (Research Grant or Support)**ViiV** (Scientific Research Study Investigator)

